# Assessment of Knowledge, Attitudes, and Practices Toward Rabies Among Community Members in and Around Bishoftu Town, Central Ethiopia

**DOI:** 10.1002/puh2.70246

**Published:** 2026-04-26

**Authors:** Fanuel Bizuayehu Yihunie, Tesfa Worku Jambare, Ashenafi Syoum, Mequanint Addisu Belete, Teshager Dubie, Gebremedhin Gebrezgabiher

**Affiliations:** ^1^ Department of Veterinary Medicine College of Veterinary Medicine and Animal Sciences, Samara University Samara Ethiopia; ^2^ Department of Veterinary Laboratory Technology College of Agriculture and Natural Resources, Debre Markos University Debre Markos Ethiopia

**Keywords:** attitudes, Bishoftu, community, Ethiopia, knowledge, practices, rabies

## Abstract

**Introduction:**

Zoonotic diseases pose a substantial burden on both animal and human health, with the impact being particularly severe in developing countries.

**Methods:**

A cross‐sectional study was conducted between November 2023 and May 2024 to assess community knowledge, attitudes, and practices (KAP) regarding rabies in and around Bishoftu town, central Ethiopia, using a pretested, structured questionnaire. Data were collected using multistage sampling techniques and analyzed using descriptive statistics and binary logistic regression.

**Results:**

Of the 384 respondents interviewed, 210 (54.7%) were male, 229 (59.6%) were aged 20–45 years, and 183 (47.7%) had a level of education above secondary school. The participants’ overall levels of KAP were 55.2% with good knowledge, 83.9% demonstrating positive attitudes, and 42.7% exhibiting good practices. A strong association was found between knowledge scores and age, family size, occupation, and living situation (*p* < 0.05). Participants aged 21–45 (AOR = 3.1, 95% CI = 1.34, 7) and 46–60 (AOR = 3.3, 95% CI = 1.1, 9.4) showed better knowledge than younger individuals (18–20 years). The study revealed that government officials exhibited lower knowledge levels (AOR = 0.2, 95% CI = 0.1–0.4). Respondents from households with 4–6 members were 52.5% less likely (AOR = 0.5, 95% CI = 0.3, 0.9) to have good knowledge about rabies compared to those from smaller families. Females were 54.6% less likely than males to exhibit favorable attitudes (AOR = 0.5, 95% CI = 0.2, 0.9). Participants with no formal education were 7.6 times more likely to demonstrate good rabies prevention practices compared to those who attended informal schools (AOR = 7.6, 95% CI = 1.2–46.3).

**Conclusion:**

The study found that although a larger proportion of participants had good knowledge and attitudes about rabies, a smaller proportion demonstrated good practices. As the study was limited to a specific area in and near Bishoftu, a broader study covering a larger region is recommended.

## Graphical abstract

The overall levels of knowledge, attitudes, and practices toward rabies in this study were 55.2%, 83.9%, and 42.7%, respectively. Only 21.7% were aware that rabies was fatal once clinical symptoms manifested. Age groups 21–45 and 46–60 have good knowledge, and government officials have lower knowledge. Females are less likely to show favorable attitudes. Unexpectedly, no formal education is linked to better practices.

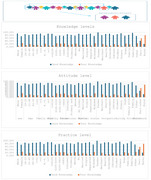

## 1 Introduction

Several zoonotic diseases impose a significant burden on both human and animal health, particularly in developing countries [1]. Among these, Neglected Tropical Zoonotic Diseases (NTZDs) disproportionately affect the world's poorest population [2]. Rabies, classified as one of the 17 major neglected tropical diseases (NTDs), is particularly concerning due to its nearly 100% fatality rate in humans once clinical symptoms appear [3].

Rabies is endemic in many countries, although it is notably absent in regions, such as Australia and Antarctica [3]. The disease affects all warm‐blooded animals and causes over 60,000 human deaths annually worldwide [4, 5]. Additionally, approximately 29 million people receive post‐exposure prophylaxis (PEP) each year to prevent rabies after potential exposure [6]. Beyond its human toll, rabies leads to substantial livestock losses, exceeding $50 million globally per year [7]. Africa bears a particularly high burden [8, 9, 10], with a disproportionate share of global rabies deaths, largely due to the role of domestic dogs as the primary source (>90%) of human infections [11]. This underscores the urgent need for improved public health interventions and vaccination programs.

In Ethiopia, rabies has been a significant public health concern for centuries [12, 13]. The first recorded rabies epidemic occurred in Addis Ababa in 1903 [13], and today, rabies remains a top‐priority zoonotic disease with a high burden and widespread distribution [14]. National surveillance data from 2007 to 2012 documented 15,178 exposure cases (3.4/100,000) and 272 fatalities, with an incidence rate of 1.5 per 100,000 [15]. Over 88% of exposures were attributed to dog bites, likely due to the large population of stray and unvaccinated dogs [16]. The high density of free‐roaming dogs, estimated at one owned dog for every five households nationwide, including both owned and stray animals, significantly contributes to disease transmission in both urban and rural areas [17].

Despite these challenges, public awareness of rabies remains inadequate, particularly in urban areas with dog populations, as evidenced by previous knowledge, attitudes, and practices (KAP) study across Ethiopia [18]. For instance, in Mekelle city, only 50% of respondents demonstrated good KAP regarding rabies prevention and control [19]. Similarly, in Asella Town, although 82% of participants were aware of rabies, only 58% recognized its zoonotic importance [20]. In Debre Tabor Town, approximately half of the respondents exhibited good KAP levels [21], whereas in Bishoftu, 61.8% had moderate knowledge, 59.8% held intermediate attitudes, and 64.3% reported satisfactory preventive practices [22].

Ethiopia has adopted a national master plan to eliminate human cases of dog‐mediated rabies by 2030. This strategy emphasizes mass dog vaccination, pre‐exposure prophylaxis and PEP, and public education to achieve rabies‐free status [23]. Effective control measures include quarantine enforcement, stray dog management, public education, wildlife rabies control, dog registration, and mass vaccination campaigns. Additionally, ensuring prompt PEP for bite victims and enhancing community awareness are critical to preventing rabies‐related deaths [24]. Investigating communities’ levels of KAP regarding rabies is essential to the design of prevention strategies, including health promotion and education for dog/pet owners, school children, and the broader public [25]. Although a prior study in Bishoftu examined dog demography, rabies awareness, and bite cases [22], its design, sample size, and analytical approach (lack of regression modeling) differed from the current study. Therefore, this study aimed to assess the KAP of community members in and around Bishoftu Town regarding rabies and to identify the factors influencing these outcomes.

## 2 Methods

### 2.1 Description of Study Area

**FIGURE 1:**
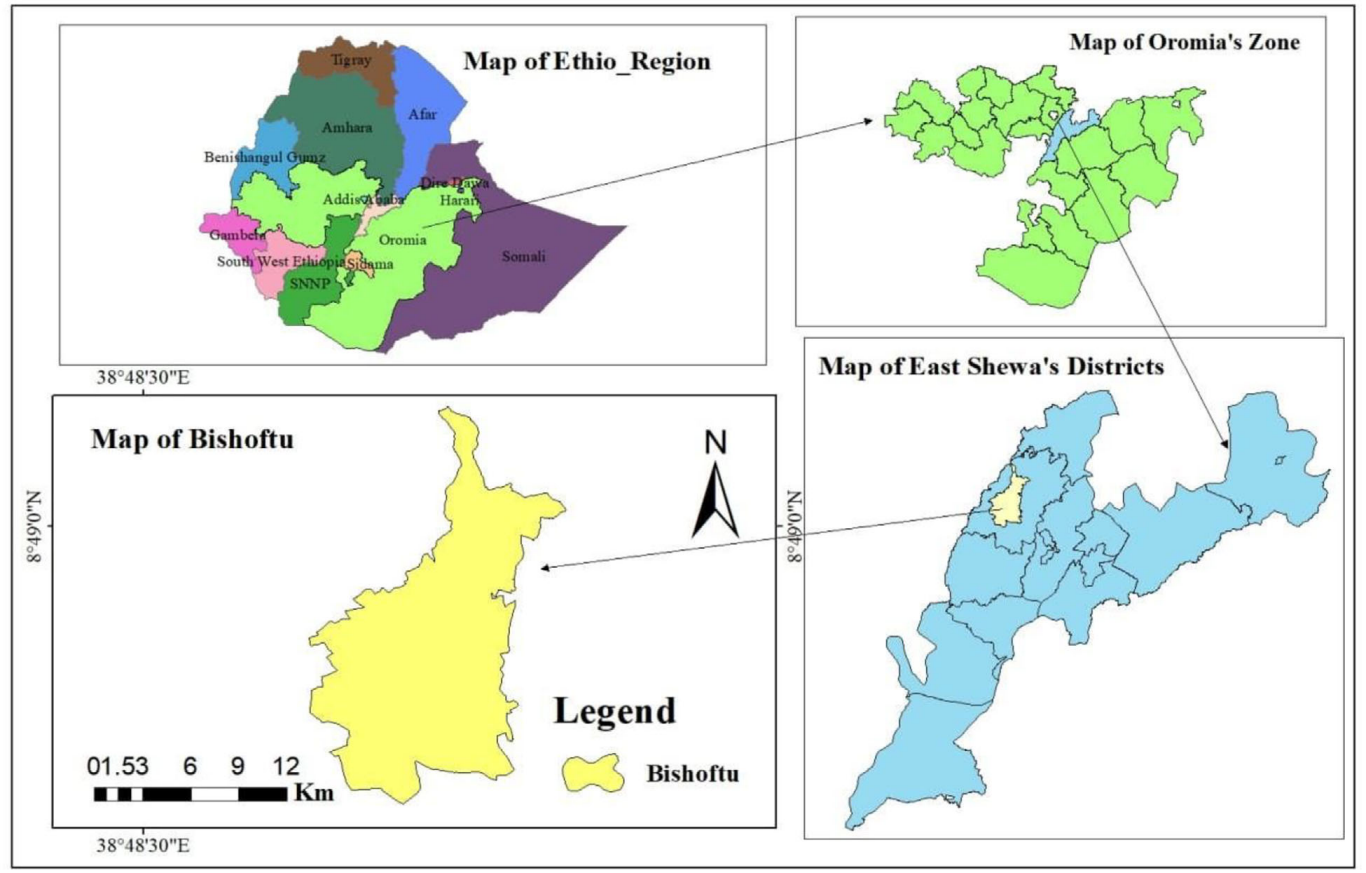
Map showing the study district in and around Bishoftu town (ArcGIS).

The study was conducted in and around Bishoftu Town, central Ethiopia. Bishoftu town is 45 km southeast of Addis Ababa (Figure 1) at 9°6′0″ N and 37°15′0″ E, with an elevation of 1850 m above sea level. The area receives an annual rainfall of 866 mm, 84% of which occurs during the long rainy season (June to September) [22]. The average temperature ranges from 12.3°C to 27.7°C. Bishoftu is divided into 14 administrative units and has a population of 127,678 residents, consisting of 59,589 men and 68,090 women. The town is encircled by numerous lakes (Bishoftu, Hora, Babogaya, Kuriftu, and Cheleklaka) and resorts (Kuriftu Resort & Spa and Liesak), attracting many nonresidents. Although the exact number of dogs in the town is lacking, there is a significant dog population (599 dog‐owning households) [22, 26]. Factors, such as urbanization, population density, and proximity to Addis Ababa, can shape rabies exposure risk and dog–human interactions in several ways. Urbanization and high population density often increase the number of free‐roaming dogs and the frequency of human–dog contact, raising the potential for bites and rabies transmission. But it may also improve access to rabies prevention resources and post‐exposure care.

### 2.2 Study Population

The study participants were household heads residing in and around Bishoftu town, representing diverse sociodemographic characteristics. The study included both male and female respondents aged above 18 years, and households that had been residents of the area for at least 6 months.

### 2.3 Study Design

A community‐based cross‐sectional study was conducted in and around Bishoftu Town, central Ethiopia, from November 2023 to May 2024. The principal aspects of the study focused on KAP about rabies transmission and prevention between animals and humans.

### 2.4 Sample Size Determination

The required sample size for this study was calculated on the basis of the assumption that 50% of respondents have good knowledge, positive attitudes, and good practices about rabies. The sample size was calculated according to Cochran's formula for categorical data [27]:

$$\begin{array}{c} n=\frac{\;t^{2}\times p\left(q\right)}{d^{2}}\\ n=\frac{1.96^{2}\times 0.5\left(1-0.5\right)}{0.05^{2}}=384 \end{array}$$

where *n* is the sample size, *t* is the value for the selected alpha level of 0.05 = 1.96, *p* is the estimate of the proportion of level of KAP present in the population, *q* is the estimate of variance = 0.5, and *d* is the acceptable margin of error = 0.05. Then, the total number of respondents was 384.

### 2.5 Sampling Technique

**FIGURE 2:**
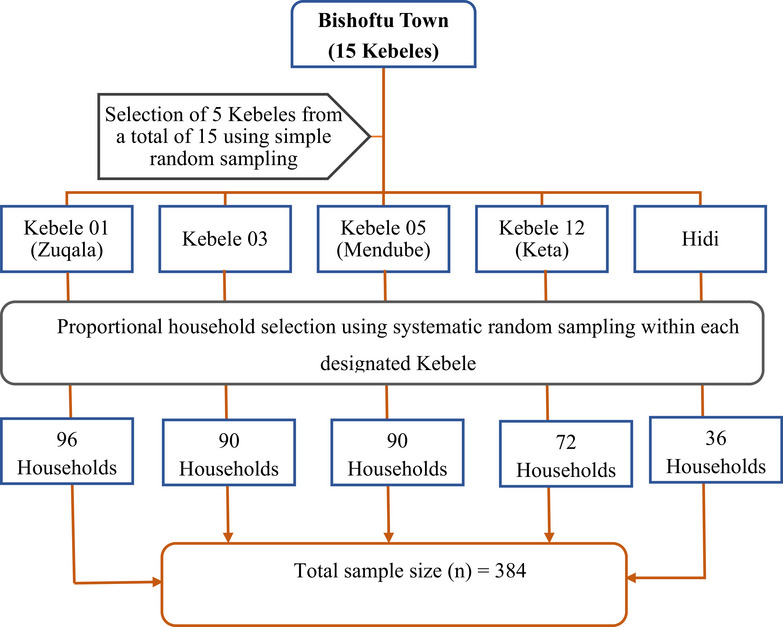
Schematic representation of sampling methods and procedures to select study participants, in Bishoftu town, Oromia region, Ethiopia, 2024.

Five Kebeles (Kebele is the smallest administrative unit in Ethiopia) from the 14 in and around Bishoftu town were selected using a simple random sampling technique. These Kebeles were Kebele 01 (Zuqala), Kebele 03, Kebele 05 (Mendube), Kebele 12 (Keta), and Hidi. The total sample size was proportionally divided among these five Kebeles based on the population size. Household heads were then selected using systematic random sampling, provided that the first household was sampled at random and the next households using a determined sampling interval. A total of 384 participants were selected as follows: Kebele 01 (96 participants), Kebele 03 (90 participants), Kebele 05 (90 participants), Kebele 12 (72 participants), and Hidi (36 participants) (Figure 2). The sample size for each Kebele was determined using the following formula [27]:

$$nj=\frac{n\;}{N}Nj$$

where *nj* is sample size of the *j*th kebele, *Nj* is the population size of the *j*th Kebele, *n* = *n*1 + *n*2 + ⋯ + *nk* is the total sample size, *N* = *N*1 + *N*2 + ⋯ + *N*k is the total population size.

### 2.6 Data Collection Method

Data were collected using a pretested, structured questionnaire. The questionnaire was developed on the basis of information gathered from various sources, and some were adopted from other studies [21, 28, 29]. Initially, the questionnaire was prepared in English, professionally translated to Afan Oromo and Amharic, and back translated to English to verify accuracy. The questionnaire was pretested using expert review and a pretest study using 10 individuals selected in the study area at random. Interviews were conducted in the participants’ preferred language. The interviews were conducted face‐to‐face by research team members with a veterinary medicine background. Respondents who had not heard about rabies would not answer the questions related to rabies. In our case, 39 respondents were not asked the following question after “Have you heard about rabies.”

### 2.7 Statistical Analysis

Thirty questions were used as follows: 16 knowledge questions (one point for a correct answer, max 16), 4 attitudes questions (maximum score of 4), and 9 practice questions that were scored out of a maximum obtainable score of 10. The total maximum score was 29 points. Data were recorded in Microsoft Excel 2024 (Microsoft Corporation, USA) and cleaned to avoid errors prior to analysis. Statistical analysis was performed using SPSS version 20. KAP scores were calculated by assigning one point for correct responses and zero for incorrect ones. Participants could select multiple options for some questions (e.g., transmission routes), with one point awarded for correct responses. Mean scores were used to assess the knowledge and practices as the data were normally distributed according to the Shapiro–Wilk test (*p* 0.05 for knowledge and practices score), whereas median scores were used for attitudes as they were not normally distributed (*p* < 0.05). Participants scoring equal to or above the mean/median were classified as having good KAP levels, whereas those below were considered to have poor levels. Descriptive statistics (frequencies and percentages) were used to summarize participants’ characteristics. Binary logistic regression was used to examine associations between KAP scores and demographic factors. We assessed multicollinearity using the variance inflation factor (VIF values between 1 and 1.67), which indicated no or acceptable multicollinearity. Univariate analysis identified factors with a *p* value ≤0.25, which were then included in the multivariable analysis to control for confounders. This threshold was chosen to prevent premature exclusion of potentially important variables. The Hosmer–Lemeshow goodness‐of‐fit test was applied to assess calibration between observed and predicted outcomes, and the model demonstrated acceptable fit. In addition, potential interaction effects between variables were examined using factor‐variable notation; however, no statistically significant interactions were identified.

## 3 Results

### 3.1 Sociodemographic Characteristics

Of the 384 respondents, 54.7% were male, and 59.6% were aged between 21 and 45 years old. Nearly half (47.7%) had education beyond the secondary level, and 49.2% were married. Additionally, 49.2% of the respondents earned a monthly income of ≤$100 (Table 1).

**TABLE 1:** Sociodemographic characteristics of the study participants.

Variable	Category	Frequency (%)
Sex	Male	210 (54.7)
	Female	174 (45.3)
Age	18–20	55 (14.3)
	21–45	229 (59.6)
	46–60	84 (21.9)
	≥61	16 (4.2)
Family size	≤3	92 (24.0)
	4–6	212 (55.7)
	≥7	78 (20.3)
Income per month (in USD)	≤100	189 (49.2)
	100–200	116 (30.2)
	200–400	69 (18.0)
	≥400	10 (2.6)
Educational status	No formal education	8 (2.1)
	Informal school	63 (16.4)
	Primary and secondary	130 (33.9)
	Above secondary	183 (47.7)
Marital status	Married	189 (49.2)
	Single	171 (44.5)
	Divorced	7 (1.8)
	Widow	17 (4.4)
Occupation	Farmer	50 (13.0)
	Merchant	63 (16.4)
	Health professional (veterinary and human)	9 (2.3)
	Government officer	83 (21.6)
	Jobless	20 (5.2)
	Student	72 (18.8)
	Others	87 (22.7)
Living situation	Dogs cohabiting with family (no separate housing)	213 (55.5)
	A family with no dogs	18 (4.7)
	A family owning dogs with separate housing	153 (39.8)
Residence	Urban	350 (91.1)
	Peri‐urban	29 (7.6)
	Rural	5 (1.3)

*Note:* Others in occupation (teachers, daily workers, and private workers).

### 3.2 General Knowledge of the Study Participants Toward Rabies

The mean value of the knowledge was 9.8. Accordingly, 55.2% of the participants had good knowledge about rabies. The majority of respondents (89.8%) had heard about rabies, most commonly referring to it by local names, such as *Ye'ebd wusha beshita* in Amharic and *dhukkuba saree maratuu* in Afan Oromo, both of which mean “mad dog disease.” Only 21.7% were aware that rabies was fatal once clinical symptoms manifested. Forty‐five‐point five percent of respondents heard about rabies from health professionals and veterinarians. A critical misconception was identified in the survey as follows: 78.3% of respondents incorrectly believed that rabies can be easily treated after the onset of clinical signs. This finding warrants explicit emphasis, as it highlights a dangerous gap in the understanding of rabies prognosis. Additionally, 66.1% of respondents referred to biting as a way of transmission, 81.4% of respondents were aware of PEP, and 93% recognized the benefits of dog vaccination in preventing rabies (Table 2).

**TABLE 2:** Knowledge of the study participants towards rabies.

Knowledge questions	Response	Frequency (%)
Have you heard about rabies?	Yes	345 (89.8)
	No	39 (10.2)
What is rabies?	Disease	183 (53.0)
	Change in behavior	140 (40.6)
	I don't know	22 (6.4)
Which species are affected by rabies?	Dog and human	87 (25.2)
	Human and other domestic animal	115 (33.3)
	Fox	2 (0.6)
	I don't know	3 (0.9)
	All	138 (40)
Rabies can be easily treated after the onset of clinical signs	Yes	270 (78.3)
	No	75 (21.7)
Which age group is most affected by rabies?	Young	37 (10.7)
	Adult	17 (4.9)
	Only old man	41 (11.9)
	All	250 (72.5)
Where did you hear about rabies?	Television	53 (15.4)
	Radio	20 (5.8)
	From health professionals	157 (45.5)
	Informally (friends, family)	115 (33.3)
What happens to humans if not given post‐exposure prophylaxis after exposure to a rabid dog bite?	Survives	28 (8.1)
	Dies	292 (84.7)
	Nothing happens	7 (2.0)
	I don't know	18 (5.2)
Which body part is most affected?	Brain	179 (51.9)
	Stomach	7 (2.0)
	Bitten area	18 (5.2)
	All parts	134 (38.9)
	I don't know	7 (2.0)
Can it be transmitted to humans?	Yes	324 (93.9)
	No	21 (6.1)
Can it be transmitted to other animals?	Yes	321 (93.0)
	No	24 (7.0)
What is the mode of transmission to humans?	Biting	228 (66.1)
	Inhalation	7 (2.0)
	Scratch	5 (1.5)
	Saliva contact with an open wound	73 (21.2)
	Living with an animal	2 (0.6)
	I don't know	30 (8.7)
Which clinical sign did you observe in rabid dogs	Aggressiveness	31 (9.0)
	Sudden change of behavior	33 (9.6)
	Loss of appetite	11 (3.2)
	Paralysis	5 (1.4)
	Difficulty sleeping	12 (3.5)
	Hydrophobia	30 (8.7)
	Salivation	51 (14.8)
	All	172 (49.8)
	I don't know	0
Do you know about post‐exposure prophylaxis?	Yes	281 (81.4)
	No	64 (18.6)
Is that possible to prevent rabies in dogs by vaccination?	Yes	321 (93.0)
	No	24 (7.0)
Do you know the vaccination interval for pets?	Yes	181 (52.5)
	No	164 (47.5)
Rabies vaccine for dogs is available in Ethiopia	Yes	326 (94.5)
	No	19 (5.5)

### 3.3 Attitudes of the Study Participants Toward Rabies

The median value used for the cutoff value of attitudes was 2. Hence, 83.9% of respondents had good attitudes toward rabies. The majority of the respondents (55.1%) believed consuming meat from a rabid animal could transmit the disease. Additionally, 78.3% respondents would go to a health care center if bitten by a rabid dog. More than half of the respondents (57.7%) believed traditional healers/spiritual ways can have solutions for rabies (Table 3).

**TABLE 3:** Attitudes of the study participants towards rabies.

Attitudes related questions	Response	Frequency (%)
Consumption of rabid animal meat could transmit rabies	Agree	190 (55.1)
	Do not agree	88 (25.5)
	Neutral	67 (19.4)
Go to a health care center if bitten by a rabid animal	Agree	270 (78.3)
	Do not agree	31 (9.0)
	Neutral	44 (12.7)
Traditional healers/spiritual ways can help recover from rabies	Agree	199 (57.7)
	Do not agree	107 (31.0)
	Neutral	39 (11.3)
Report a stray animal bite to local authorities/concerned bodies	Agree	334 (96.8)
	Do not agree	11 (3.2)
	Neutral	0

### 3.4 Practices of the Study Participants Toward Rabies

The mean value for practices was 6.09. Of the overall respondents, 42.7% demonstrated good practices in relation to rabies in humans and animals. Although relatively high proportions of respondents demonstrated good knowledge and attitudes, fewer respondents have good practices. This sharp discrepancy highlights a critical gap between awareness and actual behavior, underscoring the need for interventions that not only improve knowledge but also translate it into consistent preventive practices. Over 69% of the respondents keep their dogs indoors. Additionally, 34.7% of respondents vaccinate their dogs against rabies. The majority, 72.1% of respondents, reported killing the rabid animals (Table 4).

**TABLE 4:** Practices of the study participants towards rabies in and around Bishoftu town, central Ethiopia.

Practices related questions	Response	Frequency (%)
How do you manage your dog?	Free to move any	100 (26.0)
	Kept indoor	266 (69.3)
	I don't have dogs	18 (4.7)
Necessary actions to be taken to keep the health of your dogs	Vaccinate regularly	127 (34.7)
	Eliminate stray dogs	51 (13.9)
	Always keep the dog tied	23 (6.3)
	All the above three options	165 (45.1)
Action to be taken on animals bitten by a rabid dog	Killing	161 (41.9)
	Treatment/vaccination	183 (47.7)
	I don't know	40 (10.4)
Action to be taken on a rabid dog	Tie	64 (16.7)
	Kill	277 (72.1)
	Nothing	43 (11.2)
Immediate action to be taken by humans after being bitten by rabid animals	Tie the wound	38 (9.9)
	Wash with water and soap	69 (18.0)
	Use a traditional healer	25 (6.5)
	Visit a health institution	213 (55.5)
	I don't know	39 (10.2)
Do you have experience on vaccination of your dog?	Yes	322 (88.0)
	No	44 (12.0)
Do you wash your hands after touching the dog?	Yes	322 (88.0)
	No	44 (12.0)
Did you ever get bitten by a dog and take PEP?	Yes	157 (40.9)
	No	227 (59.1)
Inform the authorities if a dog bites you	Yes	356 (92.7)
	No	28 (7.3)

Abbreviation: PEP, post‐exposure prophylaxis.

### 3.5 Factors Associated With Community Levels of KAP Toward Rabies

The multivariable analysis revealed that age, occupation, family size, and living situation were significantly associated with knowledge about rabies, with a 95% level of significance (*p* ≤ 0.05) (Table 5).

**TABLE 5:** Factors associated with knowledge of participants towards rabies in and around Bishoftu town, central Ethiopia.

Variables	Knowledge level		Univariable logistic regression		Multivariable logistic regression	
	Good (%)	Poor (%)	*p* value	COR (95% CI)	*p* value	AOR (95% CI)
Sex						
Male	108 (57.1)	81 (42.9)	Ref			
Female	83 (53.2)	73 (46.8)	0.402	0.8 (0.562, 1.260)		
Age						
18–20	15 (30.6)	34 (69.4)	Ref		Ref	
21–45	124 (60.2)	82 (39.8)	<0.001	3.4 (1.805, 6.366)	0.008	3.1 (1.337, 7.012)
46–60	46 (60.5)	30 (39.5)	0.001	3.5 (1.681, 7.099)	0.026	3.3 (1.150, 9.452)
≥61	5 (35.7)	9 (64.3)	0.621	1.3 (0.419, 4.289)	0.259	2.6 (0.499, 13.195)
Family size						
≤3	53 (63.6)	30 (36.4)	Ref		Ref	
4–6	94 (49.0)	97 (51.0)	0.016	0.5 (0.326, 0.891)	0.015	0.5 (0.261, 0.866)
≥7	44 (62.9)	27 (37.1)	0.727	0.9 (0.479, 1.671)	0.539	0.8 (0.379, 1.661)
Monthly income						
≤$100	82 (48.2)	88 (51.8)	Ref		Ref	
$100–$200	65 (62.5)	39 (37.5)	0.018	1.8 (1.100, 2.823)	0.052	1.9 (0.993, 3.971)
$200–$400	37 (59.7)	25 (40.3)	0.110	1.6 (0.902, 2.757)	0.725	1.2 (0.490, 2.786)
>$400	7 (77.8)	2 (22.2)	0.069	4.3 (0.891, 20.82)	0.757	1.4 (0.185, 10.131)
Educational status						
Informal school	32 (56.1)	25 (43.9)	Ref		Ref	
Primary and secondary	50 (42.7)	67 (57.3)	0.105	0.6 (0.330, 1.110)	0.348	0.7 (0.273, 1.581)
Above secondary	103 (62.8)	61 (37.2)	0.307	1.4 (0.757, 2.417)	0.172	1.9 (0.755, 4.832)
No formal education	5 (71.4)	2 (28.6)	0.306	2.4 (0.449, 12.82)	0.538	1.9 (0.244, 14.962)
Marital status						
Married	98 (57.6)	72 (42.4)	Ref		Ref	
Single	82 (53.2)	72 (46.8)	0.396	0.8 (0.550, 1.266)	0.789	1.1 (0.613, 1.903)
Separated	5 (71.4)	1 (18.6)	0.174	4.4 (0.520, 37.30)	0.652	1.7 (0.158, 19.133)
Widowed	5 (33.3)	10 (66.7)	0.083	0.4 (0.142, 1.128)	0.406	0.6 (0.138, 2.231)
Occupation						
Farmer	32 (71.1)	13 (28.9)	Ref		Ref	
Merchant	40 (71.4)	16 (28.6)	0.947	0.9 (0.426, 2.217)	0.987	0.9 (0.357, 2.750)
Health professional	6 (75.0)	2 (25.0)	0.720	1.4 (0.252, 7.365)	0.952	0.9 (0.151, 5.913)
Government officer	35 (46.7)	40 (53.3)	0.006	0.3 (0.162, 0.732)	0.000	0.2 (0.059, 0.435)
Jobless	4 (22.2)	14 (77.8)	<0.001	0.1 (0.028, 0.342)	0.003	0.1 (0.028, 0.481)
Student	27 (41.5)	38 (58.5)	0.001	0.3 (0.128, 0.603)	0.692	0.8 (0.307, 2.191)
Others	32 (41.0)	46 (58.9)	0.119	0.6 (0.260, 1.167)	0.060	0.4 (0.186, 1.036)
Living situation						
Dogs cohabiting with family (no separate housing)	108 (56.3)	84 (43.7)	Ref		Ref	
Family with no dogs	12 (80.0)	3 (20.0)	0.087	2.7 (0.864, 8.513)	0.213	2.4 (0.609, 9.311)
Family owning Dogs with separate housing	69 (50.0)	69 (50.0)	0.277	0.8 (0.510, 1.212)	0.050	0.6 (0.346, 0.99)
Residence						
Urban	173 (55.1)	141 (44.9)	Ref			
Peri‐urban	14 (53.8)	12 (46.2)	0.998	1.0 (0.467, 2.144)		
Rural	3 (60)	2 (40)	0.829	1.2 (0.201, 7.393)		

Abbreviations: AOR, adjusted odds ratio; COR, crude odds ratio; Ref, reference.

The multivariable analysis revealed that only sex had a statistically significant association with attitudes about rabies, with a 95% confidence interval (*p* ≤ 0.05) (Table 6).

**TABLE 6:** Factors associated with attitudes of participants towards rabies in and around Bishoftu town, central Ethiopia.

Variables	Attitudes level		Univariable logistic regression		Multivariable logistic regression	
	Good (%)	Poor (%)	*p* value	COR (95% CI)	*p* value	AOR (95% CI)
Sex						
Male	174 (92.1)	15 (7.9)	Ref		Ref	
Female	115 (73.7)	41 (26.3)	<0.001	0.3 (0.138, 0.460)	0.030	0.5 (0.223, 0.925)
Age						
18–20	43 (87.8)	6 (12.2)	Ref			
21–45	162 (81.8)	36 (18.2)	0.325	0.6 (0.275, 1.535)		
46–60	65 (86.7)	10 (13.3)	0.950	0.9 (0.351, 2.671)		
≥61	20 (87.0)	3 (13.0)	0.981	1.0 (0.190, 5.480)		
Family size						
≤3	76 (91.6)	7 (8.4)	Ref		Ref	
4–6	150 (78.1)	42 (21.9)	0.006	0.3 (0.149, 0.728)	0.158	0.5 (0.223, 1.276)
≥7	64 (91.4)	6 (8.6)	0.813	1.1 (0.379, 3.448)	0.706	1.3 (0.383, 4.133)
Monthly income						
≤$100	157 (90.2)	17 (9.8)	Ref		Ref	
$100–$200	80 (74.8)	27 (25.2)	<0.001	0.3 (0.166, 0.600)	0.179	0.5 (0.208, 1.340)
$200–$400	50 (78.1)	14 (21.9)	0.011	0.4 (0.179, 0.803)	0.800	0.9 (0.273, 2.725)
Educational status						
Informal school	44 (75.9)	14 (24.1)	Ref		Ref	
Primary and secondary	104 (87.4)	15 (12.6)	0.045	2.2 (1.020, 4.862)	0.631	1.3 (0.483, 3.318)
Above secondary	139 (82.7)	29 (17.3)	0.230	1.5 (0.763, 3.075)	0.550	1.3 (0.516, 3.468)
Marital status						
Married	132 (77.6)	38 (22.4)	Ref		Ref	
Single	139 (90.2)	15 (9.8)	0.002	2.6 (1.410, 4.749)	0.178	1.7 (0.789, 3.605)
Separated	5 (83.3)	1 (16.7)	0.622	1.7 (0.201, 14.638)	0.647	0.6 (0.048, 6.571)
Widowed	13 (86.7)	2 (13.3)	0.324	2.1 (0.471, 9.746)	0.941	0.9 (0.139, 6.256)
Occupation						
Farmer	41 (91.1)	4 (8.9)	Ref		Ref	
Merchant	42 (75.0)	14 (25.0)	0.022	0.3 (0.079, 0.822)	0.2	0.4 (0.089, 1.462)
Health professional	8 (100)	0 (0)	0.999	1.4 (0.002, 1.0322)	0.999	1.8 (0.001, 1.08)
Government officer	60 (81.1)	14 (18.9)	0.087	0.4 (0.114, 1.159)	0.635	0.7 (0.173, 2.915)
Jobless	16 (88.9)	2 (11.1)	0.788	0.8 (0.132, 4.653)	0.660	1.1 (0.129, 8.608)
Student	60 (90.9)	6 (9.1)	0.744	0.8 (0.223, 2.919)	0.539	0.6 (0.133, 2.874)
Others	63 (80.8)	15 (19.2)	0.080	0.4 (0.113, 1.132)	0.495	0.6 (0.170, 2.356)
Living situation						
Dogs cohabiting with family (no separate housing)	167 (87.4)	24 (12.6)	Ref		Ref	
Family with no dogs	15 (93.8)	1 (6.2)	0.389	2.5 (0.316, 19.30)	0.836	1.3 (0.126, 12.960)
A family owning dogs with separate housing	106 (76.8)	32 (23.2)	0.008	0.5 (0.265, 0.823)	0.175	0.6 (0.328, 1.224)
Residence						
Urban	261 (82.9)	54 (17.1)	Ref			
Peri‐urban	25 (96.2)	1 (3.8)	0.087	5.8 (0.773, 43.406)	0.207	3.9 (0.466, 34.005)
Rural	3 (75.0)	1 (25.0)	0.867	0.8 (0.091, 7.536)	0.347	0.3 (0.017, 4.227)

The multivariable analysis results indicate that family size, educational status, occupation, and living situation are statistically associated with rabies prevention practices (Table 7).

**TABLE 7:** Factors associated with the practices of participants towards rabies in and around Bishoftu town, central Ethiopia.

Variables	Practices level		Univariable logistic regression		Multivariable logistic regression	
	Good (%)	Poor (%)	*p* value	COR (95% CI)	*p* value	AOR (95% CI)
Sex						
Male	86 (41)	124 (59)	Ref			
Female	78 (44.8)	96 (55.2)	0.445	1.2 (0.781, 1.758)		
Age						
18–20	23 (41.8)	32 (58.2)	Ref		Ref	
21–45	100 (43.6)	129 (56.4)	0.804	1.1 (0.594, 1.957)	0.798	0.9 (0.410, 1.988)
46–60	38 (45.2)	46 (54.8)	0.691	1.1 (0.578, 2.284)	0.533	1.4 (0.505, 3.741)
≥61	3 (18.7)	13 (81.3)	0.103	0.3 (0.082, 1.257)	0.236	0.3 (0.060, 2.001)
Family size						
≤3	31 (33.7)	61 (66.3)	Ref			
4–6	97 (45.3)	117 (54.7)	0.060	1.6 (0.980, 2.715)	0.041	1.8 (1.025, 3.318)
≥7	36 (46.2)	42 (53.8)	0.099	1.7 (0.907, 3.137)	0.083	1.9 (0.920, 3.888)
Monthly income						
≤$100	78 (41.2)	111 (58.8)	Ref		Ref	
$100–$200	56 (48.3)	60 (51.7)	0.232	1.3 (0.834, 2.115)	0.831	0.9 (0.504, 1.735)
$200–$400	22 (31.8)	47 (68.2)	0.172	0.7 (0.372, 1.194)	0.388	0.7 (0.301, 1.593)
>$400	8 (80)	2 (20)	0.031	5.7 (1.177, 27.534)	0.168	3.8 (0.572, 24.693)
Educational status						
Informal school	18 (28.5)	45 (71.5)	Ref		Ref	
Primary and secondary	61 (46.9)	69 (53.1)	0.016	2.2 (1.158, 4.217)	0.015	2.9 (1.231, 6.998)
Above secondary	81 (44.3)	102 (55.7)	0.030	1.9 (1.068, 3.689)	0.020	2.9 (1.183, 7.228)
No formal education	4 (50)	4 (50)	0.228	2.5 (0.564, 11.091)	0.030	7.5 (1.220, 46.300)
Marital status						
Married	87 (46)	102 (54)	Ref			
Single	69 (40.3)	102 (59.7)	0.278	0.8 (0.522, 1.205)		
Separated	2 (28.8)	5 (71.2)	0.373	0.5 (0.089, 2.478)		
Widowed	6 (35.5)	11 (64.5)	0.397	0.6 (0.227, 1.800)		
Occupation						
Farmer	26 (52)	24 (48)	Ref		Ref	
Merchant	25 (39.6)	58 (60.4)	0.192	0.6 (0.287, 1.286)	0.037	0.4 (0.153, 0.946)
Health professional	4 (44.5)	5 (55.5)	0.677	0.7 (0.177, 3.077)	0.446	0.6 (0.118, 2.566)
Government officer	35 (42.2)	48 (57.7)	0.271	0.7 (0.332, 1.363)	0.118	0.5 (0.206, 1.194)
Jobless	6 (30)	14 (70)	0.100	0.4 (0.131, 1.195)	0.312	0.5 (0.145, 1.852)
Student	30 (41.7)	42 (58.3)	0.261	0.7 (0.319, 1.363)	0.032	0.4 (0.147, 0.916)
Others	38 (43.6)	49 (56.4)	0.348	0.7 (0.356, 1.439)	0.176	0.6 (0.274, 1.268)
Living situation						
Dogs cohabiting with family (no separate housing)	78 (36.6)	135 (63.4)	Ref		Ref	
Family with no dogs	3 (16.7)	15 (83.3)	0.102	0.3 (0.097, 1.233)	0.040	0.2 (0.061, 0.949)
Family owning dogs with separate housing	89 (58.2)	64 (41.8)	<0.001	2.4 (1.570, 3.797)	0.000	2.8 (1.677, 4.550)
Residence						
Urban	151 (43.1)	199 (56.9)	Ref			
Peri‐urban	12 (41.4)	17 (58.6)	0.854	0.9 (0.431, 2.006)		
Rural	1 (20)	4 (80)	0.323	0.3 (0.036, 2.978)		

## 4 Discussion

This study provides important insights into the community KAP regarding rabies in Bishoftu, Ethiopia. Although the findings demonstrate encouraging levels of basic awareness, they also reveal critical gaps that require urgent public health attention. Unlike prior KAP studies on rabies conducted in Ethiopia, our work specifically focused on different community members together. This provides new insights into gaps between awareness, attitudes, and practices that have not been systematically documented before. This study found that 55.2% of participants possessed good knowledge about rabies, with 89.8% having heard of the disease. Many participants believed rabies could be treated after symptom onset, revealing dangerous misunderstandings. Although the current knowledge levels were similar to those in Mekelle city, Ethiopia [19], it differed from the study in Bishoftu [22] (61.8% moderate knowledge). This variation may reflect differences in (1) scoring systems (our 16‐point scale vs. 15‐point scale), (2) sampling frames (we included peri‐urban areas), (3) both studies were using different questionnaires, and (4) Worku et al. divided the respondents into three levels. However, only 21.7% recognized the invariably fatal nature of rabies after the onset of symptoms, indicating a dangerous gap in understanding the disease's severity. Failure to recognize the invariably fatal nature of rabies once symptoms appear undermines the urgency of timely PEP and could contribute to delayed or inappropriate care‐seeking. By explicitly flagging this result, we underscore the need for intensified community education that integrates both preventive measures and the fatal prognosis of untreated rabies. This finding mirrors similar knowledge deficits observed in Uganda [30].

Knowledge levels varied significantly by demographic factors as follows: age, family size, occupation, and living situation. Participants aged 21–45 (AOR = 3.1; 95% CI: 1.34, 7) and 46–60 (AOR = 3.3; 95% CI: 1.1, 9.4) showed better understanding than younger individuals (18–20 years), likely due to greater life experience and health information exposure [31]. Interestingly, in contrast to a study in Tanzania [32], government officials in this study demonstrated lower knowledge compared to farmers (AOR = 0.2; 95% CI: 0.1, 0.4), possibly because farmers relied on dogs for security, which fosters informal learning. Unemployed individuals had markedly lower knowledge (AOR = 0.1; 95% CI: 0.03, 0.5), emphasizing the role of occupational engagement in health literacy. This finding aligns with studies in Mekelle, Ethiopia [19], and Nepal [33].

Family size was also a significant factor: Respondents from households with 4–6 members were 52.5% less likely (AOR = 0.5; 95% CI: 0.3, 0.9) to have good knowledge about rabies compared to those from smaller families (fewer than three or fewer members). This finding is consistent with a study in Western India [34].

Contrary to findings from Bharatpur, Chitwan, Nepal [35], and Jima Town [36], the participants, who kept dogs in separate housing, were 40% less likely (AOR = 0.6; 95% CI: 0.4, 0.99) to have good knowledge about rabies compared to those who did not. This finding aligns with results from Mekelle city [19], possibly due to the role of family‐based information sharing in increasing collective awareness about the disease.

Less than half of the participants knew about the clinical signs and symptoms of rabies, consistent with a study in Uganda [30]. Respondents were aware of PEP or vaccination for preventing the disease, supported by various studies [5, 37, 38, 39]. Additionally, 56.2% of the respondents knew that rabies is transmitted through the bite of rabid dogs, a finding supported by different studies [9, 40, 41, 42, 43].

In this study, 83.9% of respondents held positive attitudes toward rabies prevention and control. However, only 19.7% believed that rabies is transmitted through the bite of a rabid dog or contact of a wound with its saliva. Additionally, 55.1% of the respondents believed that consuming the meat of a rabid animal could lead to infection, indicating persistent misconceptions about the transmission routes. A majority (84.7%) believed that individuals exposed to rabies would inevitably die, a perception supported by findings from a similar study in Bangladesh [44]. Notably, 78.3% of respondents believed that treatment is possible after the onset of the clinical symptoms, likely due to the respondents’ belief that rabies can be treated traditionally. Many (57.7%) also reported that traditional healers could prepare herbal and animal‐based remedies, and some mentioned the use of spiritual rituals to cure rabies. However, there are no scientifically validated plant species or traditional remedies that can prevent or treat rabies once symptoms have appeared [45]. Such beliefs may delay timely medical interventions, leading to fatal outcomes.

Consistent with previous studies conducted in Tanzania [32] and Jimma town, Ethiopia [36], this study found that attitudes toward rabies were strikingly influenced by the gender of the respondents (*p* ≤ 0.05). Females were 54.6% less likely than males to exhibit favorable attitudes (AOR = 0.5; 95% CI: 0.2, 0.9). This disparity may reflect cultural norms limiting women's access to information, education, or participation in decision making. However, this contrasts with another study conducted in South Gondar, North West Ethiopia [21]. Such variations may be attributed to regional variations in social dynamics, public health messaging, or dog ownership practices. In some areas, men may have greater involvement with dogs, particularly for roles such as hunting and guarding, which could contribute to more informed or positive attitudes toward rabies and its management.

In this study, 42.7% of the participants demonstrated good prevention practices. Notably, 61.7% reported that they would visit a health facility after being bitten by a suspected rabid dog, whereas only 20% indicated they would immediately wash the wound with soap and water, finding that aligned with a study conducted in Uganda [43]. Additionally, 46.7% of respondents preferred to kill animals bitten by suspected rabid dogs. These actions are similar to practices observed in Philippines [24] but differ from those reported in Indonesia [5]. Additionally, 46.7% of survey participants preferred killing animals that have been bitten by rabid dogs. They also kill the rabid dogs immediately when symptoms appear, similar to practices observed in Philippines but unlike those in Indonesia.

In this study, practice levels were significantly associated with educational status, occupation, and living situation (*p* ≤ 0.05). Interestingly, no formal education participants were 7.6 times more likely to demonstrate good rabies prevention practices compared to those who attended informal school (AOR = 7.6; 95% CI: 1.2, 46.3), which contrasts with other previous findings [17]. This unexpected result may be explained by the strong culture of community engagement in Ethiopia, where experiential knowledge and social learning often shape practical behaviors. It may also be associated with potential confounding, measurement error, or contextual factors. The variation between the two Ethiopian studies could stem from geographic, lifestyle, or methodological differences. Additionally, respondents with education levels above secondary school were 2.9 times more likely to exhibit good practices compared to those with informal schooling (AOR = 2.9; 95%CI: 1.2, 7.2), aligning with previous findings by [18]. This indicates that formal education plays a critical role in promoting effective rabies prevention and control behaviors.

Unlike findings from studies conducted in Mekelle city [19] and Tanzania [32], this study found that respondents living with dogs were 2.8 times more likely to practice good rabies prevention practices (AOR = 2.8; 95% CI: 1.7, 4.6) compared to those living only with family members. Conversely, individuals living alone were 93.9% less likely to exhibit good practices (AOR = 0.2; 95% CI: 0.1, 0.9), consistent with the finding from Nepal [35]. These results highlight the importance of daily interactions with dogs and the role of communal knowledge in fostering awareness of dogs’ care and rabies prevention.

### 4.1 Limitations of the Study

One of the primary limitations of this study is its cross‐sectional nature, as data were collected at a single point in time. This restricts the ability to observe trends or changes in participants’ KAP over time. Additionally, the study was conducted exclusively in Bishoftu, Ethiopia, a specific geographic and socioeconomic setting. As such, the findings may not be fully generalizable to other areas, particularly with different cultural norms, health infrastructure, or economic conditions. Caution should therefore be taken when applying these results to broader or more diverse populations.

## 5 Conclusion

This study highlights the variability in community KAP regarding rabies, influenced by multiple sociodemographic factors. Although respondents had demonstrated good awareness of rabies and its prevention, gaps remain in understanding clinical symptoms and transmission modes, contributing to misconceptions. Age, occupation, and family size significantly influenced knowledge levels (higher in older individuals); gender affected attitudes, whereas education and living conditions correlated with practices. As the study was limited to a specific area in and near Bishoftu, a broader study covering a larger region is recommended.

## Author Contributions

**Fanuel Bizuayehu Yihunie**: conceptualization, data curation, formal analysis, investigation, methodology, resources, supervision, visualization, writing – original draft, writing – review and editing. **Tesfa Worku Jambare**: conceptualization, formal analysis, investigation, methodology, resources, supervision, writing – original draft, writing – review and editing. **Ashenafi Syoum**: conceptualization, data curation, formal analysis, investigation, methodology, writing – review and editing. **Mequanint Addisu Belete**: conceptualization, formal analysis, investigation, methodology, validation, writing – review and editing. **Teshager Dubie**: conceptualization, investigation, methodology, validation, writing – review and editing. **Gebremedhin Gebrezgabiher**: conceptualization, formal analysis, investigation, methodology, validation, writing – review and editing.

## Funding

The authors have nothing to report.

## Ethics Statement

This study was approved by the ethics review committee of Samara University (SU/IRC/06/2023). The studies were conducted in accordance with the local legislation and institutional requirements.

## Consent

A detailed oral explanation of the study protocol was provided to all participants, and written informed consent was obtained. Verbal consent was obtained from the participants of this study.

## Conflicts of Interest

The authors declare no conflicts of interest.

## Data Availability

The data that support the findings of this study are available from the corresponding author upon reasonable request.

## References

[1] L. P. N. Z. Id , D. Charypkhan , S. Hartnack , P. R. Torgerson , and S. R. Ru , “The Dual Burden of Animal and Human Zoonoses: A Systematic Review,” PLoS Neglected Tropical Diseases 1490 (2022): 1–18, 10.1371/journal.pntd.0010540. PMC9605338 36240240

[2] C. Di Bari , N. Venkateswaran , C. Fastl , et al., “The Global Burden of Neglected Zoonotic Diseases: Current State of Evidence,” One Health 17 (2023): 100595, 10.1016/j.onehlt.2023.100595. 37545541 PMC10400928

[3] C. E. Rupprecht , R. S. Mani , P. P. Mshelbwala , S. E. Recuenco , and M. P. Ward , “Rabies in the Tropics,” Current Tropical Medicine Reports 9, no. 1 (2022): 28–39, 10.1007/s40475-022-00257-6. 35371908 PMC8960221

[4] S. Kebede , G. Beyene , B. Akalu , E. Abajebel , and A. K. Id , “General Public Knowledge, Attitudes, and Practices About Rabies and Associated Factors in Gomma District of Jimma Zone, Southwestern Ethiopia,” PLoS Neglected Tropical Diseases 134 (2024): 1–19, 10.1371/journal.pntd.0012551. PMC11472934 39401195

[5] S. Rehman , F. A. Rantam , A. Rehman , M. H. Effendi , and A. Shehzad , “Knowledge, Attitudes, and Practices Toward Rabies in Three Provinces of Indonesia,” Veterinary World 14, no. 9 (2021): 2518–2526, 10.14202/vetworld.2021.2518-2526. 34840473 PMC8613781

[6] WHO , Rabies (WHO, 2024), https://www.who.int/news‐room/fact‐sheets/detail/rabies.

[7] E. G. Pieracci , B. Schroeder , A. Mengistu , et al., “Assessment of Health Facilities for Control of Canine Rabies—Gondar City, Amhara Region, Ethiopia, 2015,” MMWR Morbidity and Mortality Weekly Report 65, no. 17 (2016): 456–457. 27149318 10.15585/mmwr.mm6517a4

[8] S. Ahmed , H. Mohammed , B. Firaol , K. Friat , and G. Desta , “Assessment of Community's Knowledge, Attitude and Practice Towards Prevention and Control of Rabies in and Around Adigrat Town, Tigray Regional State,” Ethiopia 3, no. 8 (2021): 2–10.

[9] P. Ntampaka , P. N. Nyaga , F. Niragire , J. K. Gathumbi , and M. Tukei , “Knowledge, Attitudes and Practices Regarding Rabies and Its Control Among Dog Owners in Kigali City, Rwanda,” PLoS ONE 14, no. 8 (2019): e0210044, 10.1371/journal.pone.0210044. 31430285 PMC6701806

[10] T. P. Scott , A. Coetzer , K. De Balogh , N. Wright , and L. H. Nel , “The Pan‐African Rabies Control Network (PARACON): A Unified Approach to Eliminating Canine Rabies in Africa,” Antiviral Research 124 (2015): 93–100, 10.1016/j.antiviral.2015.10.002. 26545712

[11] D. L. Knobel , T. Lembo , S. E. Townsend , and S. Cleaveland , “Dog Rabies and Its Control,” in Rabies, 3rd ed. (Elsevier Inc., 2013), 10.1016/B978-0-12-396547-9.00017-1.

[12] M. Fekadu , “Rabies in Ethiopia,” American Journal of Epidemiology 115, no. 2 (1982): 266–273, 10.1093/oxfordjournals.aje.a113298. 7058785

[13] R. Pankhurst , “The History and Traditional Treatment of Rabies in Ethiopia,” Medical History 14, no. 4 (1970): 378–389, 10.1017/S0025727300015829. 5500191 PMC1034085

[14] G. B. Asfaw , A. Abagero , A. Addissie , et al., “Epidemiology of Suspected Rabies Cases in Ethiopia: 2018–2022,” One Health Advances 2, no. 3 (2024): 1–11, 10.1186/s44280-023-00036-6.

[15] EPHI , Ethiopian National Rabies Baseline Survey (EPHI, 2012).

[16] T. Haftom , “Human Rabies Surveillance in Ethiopia,” in Proceedings of the National Workshop on Rabies Prevention and Control in Ethiopia (2012).

[17] A. Bahiru , W. Molla , L. Yizengaw , S. A. Mekonnen , and W. T. Jemberu , “Knowledge, Attitude and Practice Related to Rabies Among Residents of Amhara Region, Ethiopia,” Heliyon 8, no. 11 (2022): 5–8, 10.1016/j.heliyon.2022.e11366. PMC9649959 36387566

[18] K. G. Addis , M. T. Bereket , G. Abebaw , and E. Yimam , “Assessment of Public Knowledge, Attitude and Practices Towards Rabies in the Community of Kombolcha, Southern Wollo, Amhara Reginal State, Ethiopia,” Journal of Public Health and Epidemiology 11, no. 1 (2019): 38–48, 10.5897/jphe2017.0965.

[19] W. G. Hagos , K. F. Muchie , G. G. Gebru , G. G. Mezgebe , K. A. Reda , and B. A. Dachew , “Assessment of Knowledge, Attitude and Practice Towards Rabies and Associated Factors Among Household Heads in Mekelle City, Ethiopia,” BMC Public Health [Electronic Resource] 20, no. 1 (2020): 1–7, 10.1186/s12889-020-8145-7. 31937297 PMC6961227

[20] A. A. Gurmu , “Assessment of Knowledge, Attitude and Practice (KAP) on Rabies Among Residents in and Around Asella Town, Arsi Zone, Ethiopia,” International Journal of Basic and Applied Virology 30, no. 1 (2024): 20–31.

[21] A. Bihon , D. Meresa , and A. Tesfaw , “Rabies: Knowledge, Attitude and Practices in and Around South Gondar, North West Ethiopia,” Diseases 8, no. 1 (2020): 2–13, 10.3390/diseases8010005. 32102458 PMC7151027

[22] H. Worku , K. Amenu , T. Kassa , et al., “Dog Demography, Rabies Awareness and Dog Bite Cases in Bishoftu Town, Ethiopia,” Ethiopian Veterinary Journal 27, no. 1 (2023): 112–142, 10.4314/evj.v27i1.6.

[23] Ministry of Health‐Ethiopia , National Rabies Control and Elmination Strategy (Ministry of Health‐Ethiopia, 2018).

[24] T. R. M. Barroga , I. S. Basitan , T. M. Lobete , et al., “Community Awareness on Rabies Prevention and Control in Bicol, Philippines: Pre‐ and Post‐Project Implementation,” Tropical Medicine and Infectious Disease 3, no. 1 (2018): 1–14, 10.3390/tropicalmed3010016. PMC6136611 30274414

[25] A. Assefa , A. Alie , S. Derso , and B. Ayele , “Assessment of Knowledge, Attitude and Practice on Rabies in and Around Debretabor, South Gondar, Northwest Ethiopia,” International Journal of Basic and Applied Virology 4, no. 1 (2015): 28–34, 10.5829/idosi.ijbav.2015.4.1.94266.

[26] D. Tegegne and A. Mengesha , “Estimation of Owned and Street Dog Population by Quesionnire Survey and Mark‐Recapture Method in Three Urban Areas: Bishoftu, Dukem and Modjo Towns,” Austin Journal of Veterinary Science & Animal Husbandry 9, no. 5 (2022): 1105.

[27] J. E. Bartlett , J. W. Kotrlik , and C. C. Higgins , “Organizational Research: Determining Appropriate Sample Size in Survey Research Appropriate Sample Size in Survey Research,” Information Technology, Learning, and Performance Journal 19, no. 1 (2001): 43–50, https://www.opalco.com/wp‐content/uploads/2014/10/Reading‐Sample‐Size1.pdf.

[28] S. T. Koruk , I. Koruk , and S. Kutlu , “Where Do We Stand in the Control of Rabies? Knowledge and Practices Among Physicians in a Health District in Turkey,” Wilderness and Environmental Medicine 22, no. 2 (2011): 151–155, 10.1016/j.wem.2010.12.008. 21440467

[29] F. Monje , J. Erume , F. N. Mwiine , H. Kazoora , and S. G. Okech , “Knowledge, Attitude and Practices About Rabies Management Among Human and Animal Health Professionals in Mbale District, Uganda,” One Health Outlook 2, no. 1 (2020): 24, 10.1186/s42522-020-00031-6. 33829139 PMC7993504

[30] C. G. K. Atuheire , J. Okwee‐Acai , M. Taremwa , et al., “Descriptive Analyses of Knowledge, Attitudes, and Practices Regarding Rabies Transmission and Prevention in Rural Communities Near Wildlife Reserves in Uganda: A One Health Cross‐Sectional Study,” Tropical Medicine and Health 52, no. 1 (2024): 48, 10.1186/s41182-024-00615-2. 39030649 PMC11264860

[31] G. Desa , D. Birasa , Y. Deneke , and D. Oljira , “Assessment of Knowledge, Attitude and Practice (KAP) of Community Toward Rabies in Medawelabu District, Bale Zone, Ethiopia,” International Journal of Research‐Granthaalayah 8, no. 3 (2020): 29–42, 10.29121/granthaalayah.v8.i3.2020.124.

[32] M. Sambo , T. Lembo , S. Cleaveland , et al., “Knowledge, Attitudes and Practices (KAP) About Rabies Prevention and Control: A Community Survey in Tanzania,” PLoS Neglected Tropical Diseases 8, no. 12 (2014): e3310, 10.1371/journal.pntd.0003310. 25473834 PMC4256472

[33] P. Pal , A. Yawongsa , T. N. Bhusal , R. Bashyal , and T. Rukkwamsuk , “Knowledge, Attitude, and Practice About Rabies Prevention and Control: A Community Survey in Nepal,” Veterinary World 14, no. 4 (2021): 923–942, 10.14202/vetworld.2021.933-942. PMC8167543 34083943

[34] H. K. Tiwari , A. T. Vanak , M. O'Dea , and I. D. Robertson , “Knowledge, Attitudes and Practices (KAP) Towards Rabies and Free‐Roaming Dogs (FRD) in Shirsuphal Village in Western India: A Community Based Cross‐Sectional Study,” PLoS Neglected Tropical Diseases 13, no. 1 (2019): e0007120, 10.1371/journal.pntd.0007120. 30682015 PMC6364945

[35] S. Subedi , K. Adhikari , D. Regmi , et al., “Assessment of Community Knowledge and Practices Towards Rabies Prevention: A Cross‐Sectional Survey in Bharatpur, Chitwan, Nepal,” Zoonotic Diseases 3, no. 3 (2023): 203–214, 10.3390/zoonoticdis3030017.

[36] T. Kabeta , B. Deresa , W. Tigre , M. P. Ward , and S. M. Mor , “Knowledge, Attitudes and Practices of Animal Bite Victims Attending an Anti‐Rabies Health Center in Jimma Town, Ethiopia,” PLoS Neglected Tropical Diseases 9, no. 6 (2015): e0003867, 10.1371/journal.pntd.0003867. 26114573 PMC4482645

[37] L. Glasgow , A. Worme , E. Keku , and M. Forde , “Knowledge, Attitudes, and Practices Regarding Rabies in Grenada,” PLoS Neglected Tropical Diseases 13, no. 1 (2019): e0007079, 10.1371/journal.pntd.0007079. 30695024 PMC6368385

[38] E. Hiby , K. K. Agustina , K. N. Atema , et al., “Dog Ecology and Rabies Knowledge of Owners and Non‐Owners in Sanur, a Sub‐District of the Indonesian Island Province of Bali,” Animals 8, no. 7 (2018): 112, 10.3390/ani8070112. 29976915 PMC6070915

[39] A. Khan , R. Ayaz , A. Mehtab , et al., “Knowledge, Attitude & Practices (KAPs) Regarding Rabies Endemicity Among the Community Members, Pakistan,” Acta Tropica 200 (2019): 105156, 10.1016/j.actatropica.2019.105156. 31491398

[40] K. Bouaddi , A. Bitar , M. Bouslikhane , A. Ferssiwi , A. Fitani , and P. P. Mshelbwala , “Knowledge, Attitudes, and Practices Regarding Rabies in El Jadida Region, Morocco,” Veterinary Sciences 7, no. 1 (2020): 29, 10.3390/vetsci7010029. 32121594 PMC7157748

[41] R. T. Digafe , L. G. Kifelew , and A. F. Mechesso , “Knowledge, Attitudes and Practices Towards Rabies: Questionnaire Survey in Rural Household Heads of Gondar Zuria District, Ethiopia,” BMC Research Notes 8, no. 1 (2015): 400, 10.1186/s13104-015-1357-8. 26328612 PMC4566865

[42] D. Li , Q. Liu , F. Chen , et al., “Knowledge, Attitudes and Practices Regarding to Rabies and Its Prevention and Control Among Bite Victims by Suspected Rabid Animals in China,” One Health 13 (2021): 100264, 10.1016/j.onehlt.2021.100264. 34036144 PMC8135036

[43] M. Omodo , M. Ar Gouilh , N. Mwiine , et al., “Rabies in Uganda: Rabies Knowledge, Attitude and Practice and Molecular Characterization of Circulating Virus Strains,” BMC Infectious Diseases 20, no. 1 (2020): 200. 32143593 10.1186/s12879-020-4934-y PMC7060555

[44] M. S. Rana , A. A. Jahan , S. M. G. Kaisar , et al., “Knowledge, Attitudes and Perceptions About Rabies Among the People in the Community, Healthcare Professionals and Veterinary Practitioners in Bangladesh,” One Health 13 (2021): 100308, 10.1016/j.onehlt.2021.100308. 34458544 PMC8379336

[45] E. A. Beasley , R. M. Wallace , A. Coetzer , L. H. Nel , and E. G. Pieracci , “Roles of Traditional Medicine and Traditional Healers for Rabies Prevention and Potential Impacts on Post‐Exposure Prophylaxis: A Literature Review,” PLoS Neglected Tropical Diseases 16, no. 1 (2022): e0010087, 10.1371/JOURNAL.PNTD.0010087. 35051178 PMC8775316

